# Response to clopidogrel is associated with early neurological deterioration after acute ischemic stroke

**DOI:** 10.18632/oncotarget.24945

**Published:** 2018-04-13

**Authors:** Xingyang Yi, Jing Lin, Yanfen Wang, Ju Zhou, Qiang Zhou, Chun Wang

**Affiliations:** ^1^ Department of Neurology, People's Hospital of Deyang City, Deyang 618000, Sichuan, China; ^2^ Department of Neurology, the Third Affiliated Hospital of Wenzhou Medical University, Wenzhou 325200, Zhejiang, China

**Keywords:** ischemic stroke, clopidogrel resistance, early neurological deterioration, sroke recurrence

## Abstract

**Purpose:**

The relationship between response to clopidogrel and early neurological deterioration (END) after acute ischemic stroke (IS) is not well defined. The aim of present study was to evaluate the associations of clopidogrel resistance (CR) with END, and stratified analyze the effectiveness of clopidogrel alone and clopidogrel plus aspirin for the prevention of END.

**Results:**

A total of 375 patients, 144 patients were received clopidogrel alone, 231 patients took clopidogrel plus aspirin. CR occurred in 153 patients (40.8%). 95 (25.3%) patients developed END within the first 10 days. Platelet aggregation was higher on admission, and inhibition of platelet aggregation was significantly lower in patients with END than patients without END. Diabetes mellitus, CR, and clopidogrel plus aspirin were independently associated with END. Dual antiplatelet therapy with aspirin and clopidogrel can inhibit both arachidonic acid (AA)-induced and ADP-induced platelet aggregation

**Methods:**

This was a prospective, two-center study. A total of 375 IS patients taking clopidogrel alone or clopidogrel plus aspirin were enrolled. Platelet aggregation was measured before and after the 7–10 day treatment. CR was assessed by adenosine diphosphate (ADP)-induced platelet aggregation. The primary endpoint was END within the 10 days after admission. The secondary endpoint was a composite of recurrent ischemic stroke, myocardial infarction, and death during the 10 days after admission.

**Conclusions:**

CR and END are fairly common after acute IS. CR is associated with higher risk of END. Clopidogrel plus aspirin combination therapy provides greater inhibition of platelet aggregation, and may afford protection against END.

## INTRODUCTION

Stroke is one of the leading causes of mortality among the elderly [1], and ischemic stroke (IS) accounts for 80% of all strokes [2]. Patients with an acute IS are at a high risk of developing an early neurological deterioration (END) and recurrent ischemic stroke (RIS) [3]. END occurs in 20% to 40% of patients with acute IS, and is associated with increased morbidity and mortality [3–6]. Once deterioration has occurred, spontaneous reversal with conservative management occurs only in one-third of these patients, a large proportion of patients who deteriorated did not recover back to predeterioration deficits [7]. However, the underlying mechanisms for END are not completely understood, although various factors associated with END have been reported [8, 9]. Thus, it is very important to underscore the importance of prediction, and target therapies to reverse, halt, or even prevent deterioration in patients with acute IS.

At the current time, antiplatelet drugs such as aspirin or clopidogrel are recommended for IS patients [10]. In patients with a history of IS, antiplatelet therapy is known to reduce the incidence of ischemic events by 22% [11]. The CAPRIE (Clopidogrel Versus Aspirin in Patients at Risk of Ischemic Events) trial demonstrated that clopidogrel was shown to be superior to aspirin in reducing IS risk in patients with stroke, myocardial infarction (MI), or peripheral vascular disease [12]. However, a proportion of patients receiving clopidogrel treatment are resistant to the effects of the clopidogrel as measured by platelet function testing. Such a situation is referred as clopidogrel resistance (CR), i.e., poor or no response to clopidogrel treatment [13]. Patients with coronary ischemia who are nonresponders to clopidogrel and aspirin are at greater risk of subsequent ischemic vascular events and death [14]. Several studies have shown that reduced response to clopidogrel ranges from 4.8–51% [15, 16]. Our previous studies showed that the incidence of CR in the Chinese population was 40%, and CR was associated with RIS during 6 months after stroke [17, 18].

Most studies on antiplatelet drug responsiveness have focused on evaluating stroke recurrence following IS, very limited studies have examined the relationship between antiplatelet drug resistance and END after acute IS. Our previous studies have shown that aspirin resistance is not only associated with RIS [19, 20], but also associated with END after acute IS [5, 21]. The association between CR and END following IS has not been specifically addressed, although our previous studies have shown CR is associated with RIS [17, 18]. Therefore, the aim of present study was to evaluate the association between CR and END, and stratified analyze the effectiveness of clopidogrel alone and clopidogrel plus aspirin for the prevention of END on the basis of our previous study [22].

## RESULTS

### Characteristics of patients and occurrence of CR in acute IS patients

The mean arachidonic acid (AA)-induced platelet aggregation was 75.7 ± 18.7% before clopidogrel treatment and 41.8 ± 12.6% after 7–10 days of treatment, the mean inhibition of platelet aggregation was 32.8 ± 12.8%, while the mean adenosine diphosphate (ADP)-induced platelet aggregation was 72.4 ± 17.2% before treatment and 33.9 ± 12.1 after treatment, the mean inhibition of platelet aggregation was 40.8 ± 13.2%.

A total of 375 enrolled patients, 144 patients were received clopidogrel alone, 231 patients (minor IS or symptomatic carotid or intracranial artery stenosis) took clopidogrel plus aspirin, 153 (40.8%) patients were CR, while 222 patients (59.2%) were clopidogrel sensitive (CS). CR was significantly associated with greater age (*p* = 0.013), diabetes mellitus (*p* < 0.001), and higher fasting plasma glucose levels (*p* < 0.001). However, there were no significant differences in other factors between CR group and CS group (all *p* > 0.05). The detailed information of the patients was shown in the Table 1 of our previous article [22].

**Table 1:** Characteristics of patients with or without END

	Patients with END (*n* = 95)	Patients without END (*n* = 280)	*p* value
Age (years)	70.2 ± 11.4	67.8 ± 13.6	0.017
Male (*n*, %)	59 (62.1)	183 (65.4)	0.562
Hypertension (*n*, %)	76 (80.0)	217 (77.5)	0.633
Diabetes mellitus (*n*, %)	51 (53.7)	106 (37.9)	0.008
Current smoker (*n*, %)	40 (42.1)	118 (42.1)	0.997
Body mass index (kg/m^2^)	24.1 ± 5.8	23.9 ± 6.3	0.883
Previous MI (*n*, %)	5 (5.3)	13 (4.6)	0.834
Hyperlipidemia (*n*, %)	67 (70.5)	195 (69.6)	0.931
Fasting blood glucose (mmol/L)	7.8 ± 2.5	6.5 ± 2.6	<0.001
Hemoglobin A1c (%)	7.5 ± 1.9	6.7 ± 2.1	<0.001
Admission NIHSS	6.1 ±1.9	5.9 ± 1.8	0.344
Stroke subtype			
Atherothrombotic (*n*, %)	59 (62.1)	172 (61.4)	0.987
Small artery disease (*n*, %)	36 (37.9)	108 (38.6)	0.987
Previous treatment (*n*, %)			
Antihypertensive drugs	39 (41.1)	124 (44.3)	0.523
Hypoglycemic drugs	38 (40.0)	102 (36.4)	0.541
Statins	15 (15.8)	49 (17.5)	0.712
Aspirin	24 (25.3)	81(28.9)	0.492
In-hospital treatment (*n*, %)			
Antihypertensive drugs	83 (87.4)	239 (85.4)	0.644
Hypoglycemic drugs	56 (58.9)	140 (50.0)	0.123
Statins	94 (98.9)	275 (98.2)	0.992
Clopidogrel plus aspirin	57 (60.0)	174 (62.1)	0.721
Clopidogrel alone	38 (40.0)	106 (37.9)	0.721

END, early neurological deterioration; MI, myocardial infarction; NIHSS, National Institutes of Health Stroke Scale.

There was also no significant difference in incidence of CR between patients receiving clopidogrel alone and those taking clopidogrel plus aspirin (40.3% [58/144] vs. 41.1% [95/231], *p* = 0.65) or between patients with atherothrombotic and small artery disease (40.7% [94/231] vs. 39.6% [57/144], *p* = 0.88).

### Clinical outcomes and association of platelet aggregation with END

Among the 375 patients, the average duration of in-hospital was 13.8 days. There were no patients discharged within 10 days after admission. Based on the defined criteria, 95 (25.3%) patients developed END, 3 (0.8%) had RIS, 2 (0.5%) died, and 1 (0.3%) had MI within the first 10 days of admission. Baseline characteristics of these patients with and without END are summarized in Table 1. Univariate analyses revealed that old age, diabetes mellitus, fasting plasma glucose, and hemoglobin A1c were associated with END (Table 1).

Compared to the patients who did not suffer END, the platelet aggregation induced by AA or ADP before and after 7–10 days of clopidogrel treatment was significantly higher, and the inhibition of platelet aggregation induced by AA or ADP was significantly lower in patients who did experience these afflictions (*P* < 0.001 for each; Table 2).

**Table 2:** Platelet aggregation before and after 7–10 days of clopidogrel treatment in patients with and without END

	Patients with END (*n* = 95)	Patients without END (*n* = 280)	*p* value
AA-induced platelet aggregation (%)			
before clopidogrel	84.2 ± 15.4	71.6 ± 18.6	<0.001
after 7–10 days	54.8 ± 11.7	35.7 ± 12.8	<0.001
inhibition	29.6 ± 8.9	37.2 ± 10.3	<0.001
ADP-induced platelet aggregation (%)			
before clopidogrel	78.5 ± 15.3	68.4 ± 13.6	<0.001
after 7–10 days	44.6 ±12.2	24.2 ± 9.4	<0.001
inhibition	34.7 ±10.6	45.3 ±11.6	<0.001

END, early neurological deterioration; AA, arachidonic acid; ADP, adenosine diphosphate.

### Association of CR with outcomes

Among the 153 patients with CR, END was found in 55 (35.9%) patients, the frequency of END was significantly higher than patients with CS (18.0%) (Table 3). However, the frequencies of RIS, MI and death did not differ significantly between the two groups (Table 3). There were also no significant differences in the rates of extracranial bleeding, asymptomatic intracerebral hemorrhage (ICH) and asymptomatic hemorrhagic transformation (HT) between the two groups (Table 3). There were no serious hemorrhage, symptomatic HT or symptomatic ICH events observed in either of the two groups of patients.

**Table 3:** Association of CR with clinical outcomes

	CR (*n* = 153)	CS (*n* = 222)	*p* value
END (*n*, %)	55 (35.9)	40 (18.0)	< 0.001
RIS (*n*, %)	1 (0.7)	2 (0.9)	0.784
MI (*n*, %)	1 (0.7)	0 (0.0)	0.223
Death (*n*, %)	1 (0.7)	1 (0.5)	0.752
Safety outcomes			
Asymptomatic HT (*n*, %)	3 (2.0)	4 (1.8)	0.992
Asymptomatic ICH (*n*, %)	1(0.7)	1 (0.5)	0.753
Extracranial bleeding (*n*, %)	4 (2.6)	7 (3.2)	0.821

CR, clopidogrel resistance; CS, clopidogrel sensitivity; END, early neurological deterioration; RIS, recurrent ischemic stroke; MI, myocardial infarction; HT, hemorrhagic transformation; ICH, intracranial hemorrhage.

### Association of antiplatelet treatment with outcomes

Among the 375 patients, 144 patients received clopidogrel alone treatment, and 231 patients received clopidogrel plus aspirin treatment. There were no significant differences in baseline clinicopathological features between patients receiving clopidogrel plus aspirin and clopidogrel alone (Table 4). The frequency of END was significantly lower in patients receiving clopidogrel plus aspirin than patients receiving clopidogrel alone (Table 4). However, there were no significant differences in the frequencies of RIS, MI, death, and hemorrhagic episodes between the two groups (Table 4).

**Table 4:** Characteristics, clinical outcomes and platelet aggregation between patients receiving clopidogrel plus aspirin and patients receiving clopidogrel alone

	clopidogrel alone (*n* = 144)	clopidogrel plus aspirin (*n* = 231)	*p* value
Age (years)	69.4 ± 10.8	68.7 ± 12.6	0.273
Male (*n*, %)	91 (63.2)	151 (65.4)	0.708
Hypertension (*n*, %)	115 (79.9)	178 (77.1)	0.512
Diabetes mellitus (*n*, %)	55 (38.2)	102 (44.2)	0.279
Current smoker (*n*, %)	60 (41.7)	98 (42.4)	0.992
Previous MI (*n*, %)	6 (4.2)	12 (5.2)	0.665
Hyperlipidemia (*n*, %)	100 (69.4)	162 (70.1)	0.989
Fasting blood glucose (mmol/L)	7.1 ± 2.6	7.3 ± 2.7	0.364
Hemoglobin A1c (%)	7.0 ± 1.7	7.1 ± 2.2	0.612
Admission NIHSS	6.1 ±1.8	5.9 ± 1.9	0.304
Stroke subtype			
Atherothrombotic (*n*, %)	82 (56.9)	149 (64.5)	0.152
Small artery disease (*n*, %)	62 (43.1)	82 (35.5)	0.152
In-hospital treatment (*n*, %)			
Antihypertensive drugs	122 (84.7)	200 (86.6)	0.621
Hypoglycemic drugs	72 (50.0)	124 (53.7)	0.482
Statins	141 (97.9)	228 (98.7)	0.991
END (*n*, %)	45 (31.3)	50 (21.6)	0.041
RIS (*n*, %)	1 (0.7)	2 (0.9)	0.982
MI (*n*, %)	0 (0.0)	1 (0.4)	0.432
Death (*n*, %)	1 (0.7)	1 (0.5)	0.723
Safety outcomes			
Asymptomatic HT (*n*, %)	2 (1.4)	5 (2.2)	0.342
Asymptomatic ICH (*n*, %)	1(0.7)	1 (0.5)	0.723
Extracranialbleeding (*n*, %)	3 (2.1)	8 (3.5)	0.424
AA-induced platelet aggregation (%)			
before clopidogrel	75.2 ± 16.9	76.1 ± 19.3	0.643
after 7–10 days clopidogrel	46.9 ± 12.4	27.2 ± 9.6	<0.001
inhibition	27.7 ± 9.6	49.2 ±12.3	<0.001
ADP-induced platelet aggregation (%)			
before clopidogrel	73.2 ± 16.7	72.1 ± 17.6	0.522
after 7–10 days clopidogrel	32.7 ± 11.8	31.8 ± 9.8	0.442
inhibition	39.9 ± 10.9	41.3 ± 11.6	0.871

MI, myocardial infarction; NIHSS, National Institutes of Health Stroke Scale; END, early neurological deterioration; RIS, recurrent ischemic stroke; MI, myocardial infarction; HT, hemorrhagic transformation; ICH, intracranial hemorrhage; AA, arachidonic acid; ADP, adenosine diphosphate.

There were no significant differences in platelet aggregation induced by AA or ADP before clopidogrel between the patients on clopidogrel alone and those taking clopidogrel plus aspirin (Table 4). After 7–10 days therapy, the AA-induced platelet aggregation was significantly higher, and the inhibition of AA-induced platelet aggregation was lower in patients receiving clopidogrel alone than patients receiving clopidogrel plus aspirin (Table 4). There was no significant difference in the inhibition of ADP-induced platelet aggregation between the two groups (Table 4). Dual antiplatelet therapy with aspirin and clopidogrel could inhibit both AA-induced platelet aggregation and ADP-induced platelet aggregation (Table 4).

### Analysis of risk factors for END

**Figure 1:**
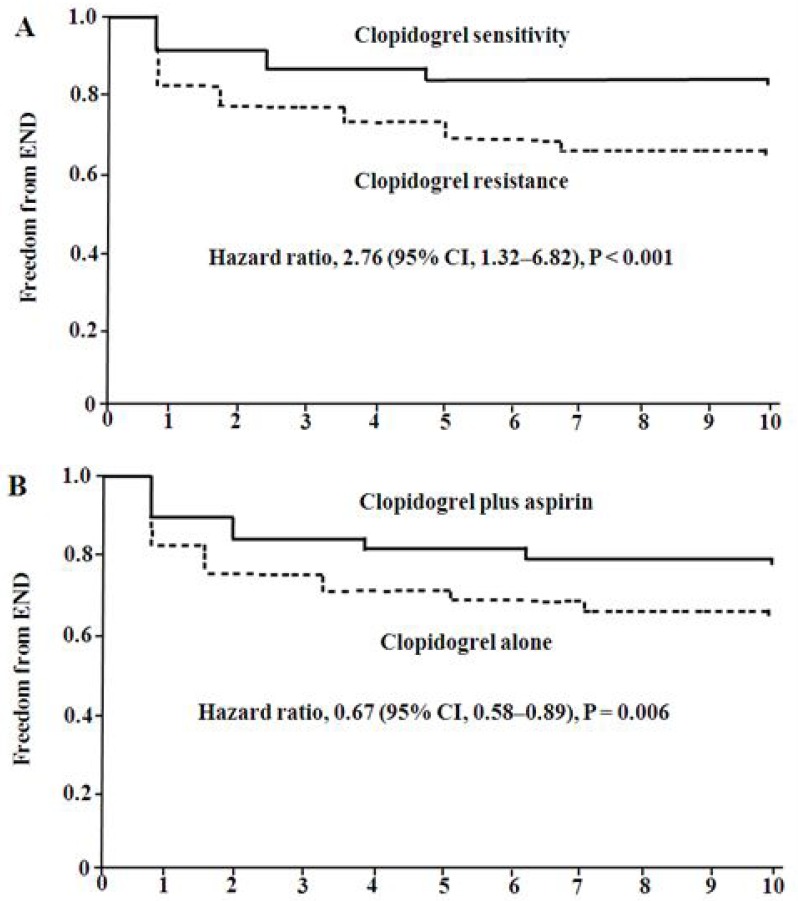
Probability of survival free of END Kaplan-Maier analysis of cumulative freedom from END associated with response to clopidogrel (**A**) and antiplatelet treatment (**B**). END indicates early neurological deterioration.

Cox proportional-hazards model was used to evaluated the risk for END. The variables that showed a significant association (*p* < 0.05) with END on univariate analysis were into the model, including age, diabetes mellitus, fasting blood glucose, hemoglobin A1C, AA-induced platelet aggregation, ADP-induced platelet aggregation, CR, and clopidogrel plus aspirin. The results showed that diabetes mellitus (hazard ratio [HR] = 1.95, 95% confidence interval [CI]: 1.04–4.67, *p* = 0.023), CR (HR = 2.76, 95% CI: 1.32–6.82, *p* < 0.001), and clopidogrel plus aspirin (HR = 0.67, 95% CI: 0.58–0.89, *p* = 0.006) were independently associated with END (Table 5). Kaplan-Meier estimates of cumulative freedom from END was significantly lower in patients with CR than patients with CS (*p* < 0.001, Figure 1A), and higher in patients receiving clopidogrel plus aspirin than patients receiving clopidogrel alone (*p* = 0.006, Figure 1B).

**Table 5:** Cox regression analysis of independent predictors for END

Factor	HR	95% CI	*P* value
Age	0.86	0.58–1.36	0.452
Diabetes mellitus	1.95	1.04–4.67	0.023
Hemoglobin A1C	1.12	0.92–2.96	0.124
Fasting blood glucose	0.93	0.78–1.79	0.383
AA-induced platelet aggregation	0.82	0.75–1.58	0.465
ADP-induced platelet aggregation	0.79	0.69–1.52	0.482
Clopidogrel plus aspirin	0.67	0.58–0.89	0.006
Clopidogrel resistance	2.76	1.32–6.82	<0.001

END, early neurological deterioration; HR, hazard ratio; CI, confidence interval.

OR for continuous variables means per 1- Standard Deviation increase.

## DISCUSSION

In this prospective, two-center observational cohort, we investigated the association of response to clopidogrel with clinical outcomes during 10 days after admission in acute IS patients receiving clopidogrel treatment. We found that 25.3% of patients developed END, 3 (0.8%) had RIS, 2 (0.5%) died, and 1 (0.3%) had MI within the first 10 days of admission. Cox regression analysis revealed that diabetes mellitus, CR, and clopidogrel plus aspirin were independently associated with END.

A number of studies have shown that END is fairly common in acute ischemic stroke [3, 5, 7–9, 21, 23, 24], and related to case fatality and reduced functional outcome [3]. The underlying mechanisms for END are not completely understood, although various factors associated with END have been reported [8, 9]. Our current results showed diabetes mellitus was one of the independent risk factors for END. The results were in accordance with our previous studies [3, 5]. Patients with diabetes mellitus showed a higher degree of platelet activation, increased expression of platelet activating binding site -1 which suggests activation of platelet glycoprotein IIb/IIIa receptor [25], and increased circulating ADP, platelet turnover and expression of P2Y12 receptors [26]. Patients with diabetes mellitus also show decreased peripheral blood flow and serum concentrations of active metabolites of clopidogrel, this may decrease antiplatelet drug responsiveness [5]. Thus, intensive antiplatelet therapy may be important in diabetic patients sustaining an IS.

Atherosclerosis and thrombosis are major causes of ischemic stroke. Platelets have a crucial role in triggering arterial thrombosis. Platelet activation has also been described in patients with IS [27]. However, it is unknown whether platelet activation is involved in the pathogenesis of END in acute IS. In present study, AA-induced or ADP-induced platelet aggregation measured both before and after 7–10 days of treatment was higher, and inhibition of AA-induced or ADP-induced platelet aggregation was lower in patients who later experienced END than in patients who did not suffer from this problem. These indicate that platelet activation may play a key role in END after acute IS. However, the pathophysiological mechanisms of platelet aggregation for END remain to be determined in future study. Some studies have reported that thrombus extension is an important cause of END [28]. Platelet activation may increase atherogenesis and promote injuries in blood vessel wall, and has a crucial role in thrombus extension [28, 29].

In this study, the most noteworthy finding was that CR was one of the important causes of END in acute IS. This suggests that insufficient inhibition of platelet activation may lead to a larger thrombus or thrombus propagation, leading to unfavorable stroke evolution [30]. Currently, despite CR signifying a risk factor for adverse events, there are no widely accepted standardized treatment recommendations for these patients. Increasing the dose of clopidogrel might reduce the rate of clopidogrel nonresponse [31], but this may increase the risk of a hemorrhagic event. Substitution of clopidogrel with another antiplatelet drug (like ticagrelor or prasugrel) is thought to another regime, and may help prevent the occurrence of vascular events [32]. Adding an additional antiplatelet agent combination therapy may be useful. The Clopidogrel in High-Risk Patients with Acute Nondisabling Cerebrovascular Events (CHANCE) trial showed that the combination of clopidogrel and aspirin for the first 21 days is superior to aspirin alone for reducing the risk of stroke in the first 90 days in patients with transient ischemic attack or minor stroke [33]. However, few studies have investigated the relationship between dual antiplatelet therapy and END after acute IS.

In present study, stratified analysis demonstrated that dual antiplatelet therapy with aspirin and clopidogrel for the first 2 weeks reduced the frequency of END more effectively, provided significantly greater inhibition of platelet activity than did the clopidogrel alone treatment. Cox proportional-hazards model showed that dual antiplatelet therapy with aspirin and clopidogrel was independently associated with END, and may afford protection against END. Antiplatelet drugs such as aspirin or clopidogrel are recommended for IS patients [10]. Our previous studies have shown that aspirin and clopidogrel can synergistically inhibit platelet aggregation because they have different pharmacological mechanisms [34, 35]. Aspirin can irreversibly inhibit cyclooxygenase, leading to decreased thromboxane A2, a platelet-aggregation activator, while clopidogrel blocks ADP to bind to its receptor on platelets to prevent clot formation. Our present results also indicate that clopidogrel can inhibit ADP-induced platelet aggregation, but do not effect on AA-induced platelet aggregation, while dual antiplatelet therapy with aspirin and clopidogrel can inhibit both AA-induced platelet aggregation and ADP-induced platelet aggregation. Thus, clopidogrel plus aspirin combination therapy for the first 2 weeks may be adequate for prevention of END in acute IS patients.

The present study has several potential limitations. First, this was a relative small size, short period of follow-up, and two-center study. Second, some studies have shown the association of biomarkers, such as high-sensitive C reaction protein, homocysteine, inflammatory cytokines, brain natriuretic peptide, and 20-Hydroxyeicosatetraenoic acid with END [3, 36]. However, these biomarkers were not measured in this study and we did not eliminate effect of these biomarkers on END. Third, although we measured platelet aggregation before and after 7–10 days of clopidogrel treatment, we did not examined plasma clopidogrel levels and its active metabolite. Fourth, the lack of a control group in the present study is a study limitation. Furthermore, although patients were received dual antiplatelet therapy with aspirin and clopidogrel or clopidogrel alone based on the guidelines, there were also no significant differences in baseline clinicopathological features between patients receiving clopidogrel plus aspirin and clopidogrel alone, it very difficult to control for selection bias. Finally, platelet aggregation was only measured using LTA in the current study. Other studies showed that vasodilator-stimulated phosphoprotein and VerifyNow P2Y12 assay could be a better choice for assessment of clopidogrel resistance [37]. Thus, future studies with a multi-center, large sample size, longer follow-up period, vasodilator-stimulated phosphoprotein and VerifyNow P2Y12 assay, and randomized-controlled trials are necessary to confirm our current findings.

## MATERIALS AND METHODS

### Study populations

This was a prospective, observational, two-center study, which was conducted in the People's Hospital of Deyang City and the Third Affiliated Hospital of Wenzhou Medical University. The study protocol was reviewed and approved by the Ethics Committees of the Third Affiliated Hospital of Wenzhou Medical University and the People's Hospital of Deyang City. Written informed consent was obtained from each participant before they were enrolled into this study. The study was registered at http://www.chictr.org/ with the unique identifier of ChiCTR-OCH-14004724.

The detailed procedures for the recruitment of IS patients, inclusion criteria and exclusion criteria were described in our previous articles [22]. Briefly, we consecutively enrolled 375 IS patients with first-time stroke, and were admitted to the participating hospitals within 72 hours of their index stroke onset between June 2014 and January 2015. In all cases, the etiology of IS was due to atherothrombotic or small artery disease according to the Trial of Org 10172 in Acute Stroke Treatment classification system [38], and National Institutes of Health Stroke Scale (NIHSS) score <15 (mild or moderate IS). Patients with cardiac cerebral embolism, other determined or undetermined etiologies of IS, or patients with platelet count <100 × 10^9^/L or >450 × 10^9^/L, and patients with thrombolytic therapy or anticoagulation therapy with warfarin or heparin within 7 days were excluded. The response rate of IS patients for the People's Hospital of Deyang City and third Affiliated Hospital of Wenzhou Medical University was 95.3% (203/213) and 96.1% (172/179), respectively. All enrolled patients received standard therapies based on standard guidelines for the prevention of stroke in patients with stroke and transient ischemic attack [10], including 75 mg clopidogrel (Sanofi Company Ltd., Beijing, China) once daily, or clopidogrel (75 mg, once daily) plus aspirin (200 mg, once daily, Bayer Healthcare Company Ltd., Beijing, China) for the initial 2 weeks, followed by treatment with clopidogrel alone (75 mg, once daily) in patients with minor IS (NIHSS score ≤3) or symptomatic carotid or intracranial artery stenosis. Data on various risk factors, including age, sex, diabetes mellitus, hypertension, current smoking, body mass index, triglycerides (TG), total plasma cholesterol (TC), low-density lipoprotein cholesterol (LDL-C), fasting plasma glucose, and hemoglobin A1C were collected. Dyslipidemia was defined as TC >200 mg/dL, TG >180 mg/dL or use of lipid-lowering medication [3].

### Study endpoints

For each patient, an NIHSS assessment was performed by a member of the stroke team upon presentation to the emergency department, and subsequently on a daily basis through the period of hospitalization. An additional NIHSS assessment was performed whenever examination deteriorated. The primary endpoint of this study was END, which was defined as an increase in NIHSS score by ≥ 2 points within 10 days after admission, while excluding a new infarct in another vascular territory or HT of infarct according to our previous studies [3, 5, 21]. The secondary endpoint was a composite of RIS, MI, and death during the first 10 days after admission. RIS was defined as a new focal neurologic deficit of vascular origin lasting for at least 24 h, which was proved to be non-hemorrhagic by either computed tomography or magnetic resonance imaging scanning. MI was defined as the presence of at least two of below criteria: prolonged angina > 30 min; electrocardiographic evidence of infarction; total creatinine kinase isoenzyme elevation more than twice the upper limit of normal. Death was defined as vascular mortality due to MI, IS, and other vascular causes. Safety endpoints included hemorrhagic episodes that occurred within 10 days after admission. Hemorrhagic episodes were defined as the presence of any of the following: (i) Symptomatic or asymptomatic HT, symptomatic or asymptomatic ICH; and (ii) Extracranial hemorrhages (e.g. gastrointestinal bleeding, hematuria, and skin or mucosal bleeding). Serious hemorrhage was considered as any symptomatic ICH or any hemorrhage requiring blood transfusion. The investigators who evaluated the clinical end-points were blinded to the results of response to clopidogrel.

### Platelet aggregation test and definition of CR

Blood samples were collected prior to the initial dose of clopidogrel and once again during day 7–10 of therapy. Platelet aggregation was measured by light transmittance aggregometry (LTA). The procedure and the consistency were assessed according to our previous studies [17–20]. In brief, the platelet aggregation test was performed using a BioData PAPS-4 platelet aggregometer (Helena Laboratories). Platelet aggregation rate was recorded as change in the light transmission. The LTA results are presented as the amplitudes of light transmittance five minutes after the addition of the agonist 0.5 mM AA and 10 μM ADP (Helena Laboratories, Beaumont, TX, USA).

According to the criteria proposed by Gurbel *et al.* [39] and our previous studies [17, 18, 22], CR was defined as a reduction of < 10% in 10.0 μM ADP-induced platelet aggregation after 7–10 days of clopidogrel treatment. All other cases (>10% reduction ADP-induced platelet aggregation) were considered as CS.

### Statistical analysis

The power and sample size of the current study were calculated by PASS (Power Analysis and Sample Size) 14.0 software (Beijing HuanZhongRuiChi Technology Co., Ltd, Beijing, China), according to assumed rate of END and CR. According to the results of Previous studies, the prevalence of END was approximately 20%–40% in acute IS patients [3–7], and 40% of the included patients were CR [17, 18]. We calculated that a minimum sample of 360 patients would provide 80% power to detect a relative risk increment of 15% in the percentage of END in the patients with CR, with a two-sided type I error of 0.05, assuming an END rate of 20% in the patients with CS.

Differences of characteristics between the patients with and without END were analyzed by univariate methods. Continuous variables are expressed as mean ± Standard Deviation and compared using Student's *t*-test if normally distributed, otherwise rank test was used. Categorical variables are presented as frequencies and percentages, and compared using Chi-square tests or Fisher's exact test if small frequencies were expected. Differences of primary endpoint, secondary endpoint and safety endpoints between the patients with CR and CS, or patients receiving clopidogrel alone and clopidogrel plus aspirin were also compared using Chi-square tests or Fisher's exact test.

Survival function estimates for END was evaluated through Kaplan-Meier analyses. Survival curves were truncated at day 10. The log-rank test was used to identify differences between patients with CR and CS, or patients receiving clopidogrel alone and clopidogrel plus aspirin. The Cox proportional-hazards model was used to describe the risk for END and reported as the HR with the 95% CI. Variables that showed a significant association (*P* < 0.05) with END on univariate analysis were into the model to adjust for confounding effects.

All statistical analyses were performed using SPSS 16.0 (SPSS Inc., Chicago, IL, USA), and all tests were two-sided. A *P* value < 0.05 was considered statistically significant.

### Ethics approval

The study protocol was approved by the Ethics Committee of the People's Hospital of Deyang City and the Third Affiliated Hospital of Wenzhou Medical University. Written informed consent was obtained from each patient prior to study enrollment.

## CONCLUSIONS

END is fairly common after acute IS in Chinese Population. Platelet activation may play a key role in END. The frequency of END was significantly higher in patients with CR than patients with CS. Diabetes mellitus, CR, and clopidogrel plus aspirin were independently associated with END. Dual antiplatelet therapy with aspirin and clopidogrel can inhibit both AA-induced and ADP-induced platelet aggregation, and may afford protection against END. However, well designed studies are needed to validate our findings in future.

## Abbreviations

END: early neurological deterioration
IS: ischemic stroke
CR: clopidogrel resistance
CS: clopidogrel sensitivity
AA: arachidonic acid
ADP: adenosine diphosphate
RIS: recurrence ischemic stroke
MI: myocardial infarction
NIHSS: National Institutes of Health Stroke Scale
TC: total cholesterol
TG: triglycerides
LDL-C: low-density lipoprotein cholesterol
LTA: light transmittance aggregometry
HT: hemorrhagic transformation
ICH: intracranial hemorrhage
HR: hazard ratio
CI: confidence interval.
